# The Need to Feel Safe: Older Adults’ and Family Caregivers’ Perspectives on Safe and Person-Centered Medication Management in Home Nursing Care

**DOI:** 10.2147/PPA.S606768

**Published:** 2026-06-20

**Authors:** Unn Siri Olsen, Erik Jakobsen, Walter Solberg, Karsten Stjern, Odd Arnevik, Gunn Eva Solum Myren, Hege Sletvold, Siri Andreassen Devik

**Affiliations:** 1 Centre for Care Research, Mid-Norway, Faculty of Nursing and Health Sciences, Nord University, Namsos, Norway; 2 Faculty of Nursing and Health Sciences, Nord University, Namsos, Norway

**Keywords:** medication safety, patient preference, qualitative interviews, co-researchers

## Abstract

**Purpose:**

To explore the experiences and preferences of older adults living at home and their family caregivers regarding medication use and medication management support through home care.

**Patients and Methods:**

Individual semi-structured interviews were conducted with 11 older adults and six family caregivers in Norway during the spring of 2024. The study employed a qualitative design involving collaboration with co-researchers. Data were analyzed using qualitative content analysis.

**Results:**

The analysis yielded three main categories—practical aspects of medication management, knowledge about the need for and effects of medications, and responsibility and collaboration—each comprising several subcategories. An overarching theme, between feeling safe and being safe: balancing autonomy and support in medication management at home, captured the balance between feeling safe as an inner sense of being cared for and being safe as external protection from harm, as well as the balance between autonomy and support in medication management. While routines and procedures in home nursing services are intended to promote safety, both older adults and family caregivers described a more passive role in medication management than they desired, often accepting what was provided while withholding questions and preferences for greater individualization. These findings indicate that achieving both feeling safe and being safe requires person-centered approaches that foster involvement, knowledge, and control.

**Conclusion:**

Person-centered medication management requires that the preferences, needs, and concerns of older adults and family caregivers are made visible and actively addressed. Strengthening communication and shared understanding between services and users is essential for achieving collaborative practice that promotes safety, autonomy, and support.

## Introduction

Medication errors are a leading cause of preventable harm globally, significantly affecting patient health outcomes.^1^ Older adults, often dealing with multiple chronic conditions and age-related physiological changes, face higher risks of adverse drug interactions and complications.^2^,^3^ Problems such as inappropriate prescribing, errors in administration, insufficient patient involvement, and discrepancies between prescribed and actual use are prevalent, especially during care transitions, including hospital admissions and discharges, where communication issues often arise.^4^ Multiple healthcare interactions and transitions often fragment medication management. Cognitive and sensory decline can further hinder medication adherence, escalating the risk of harm.^5^ Medication errors are common in home care—up to 30% in some studies—and can threaten patient safety.^6^,^7^

The older adult population is expanding, with the World Health Organization projecting that the proportion of individuals aged ≥60 years will rise from 12% in 2015 to 22% by 2050.^3^ Aging in place remains a key goal in health policy.^8^ The Decade of Healthy Ageing (2021–2030) highlights the value of person-centered and integrated care for older adults.^8^ Such care models prioritize dialogue, shared decision-making, and respect for autonomy, affirming older adults’ rights to make informed decisions about their treatment.^9^ McCormack and McCance defined person-centered practice as

…an approach to practice established through the formation and fostering of healthful relationships between all care providers, service users and others significant to them in their lives. It is underpinned by values of respect for persons, individual right to self-determination, mutual respect and understanding.^9^ (pp44–46)

In medication management, person-centeredness means viewing the person as more than just a condition or set of symptoms to be treated. In a person-centered approach, the patient is placed before the diagnosis, and dignity, autonomy, and quality of life are addressed—not just symptoms.^10^ Despite the emphasis on person-centered care, older adults are often passive in medication decision-making.^11^,^12^ Most research on medication safety focuses on the healthcare systems perspective and emphasizes quantitative outcomes such as adherence and errors, with less focus on patients’ lived experiences.^13^ Recent findings also reveal a gap between older adults’ and caregivers’ desired and actual participation levels.^14–16^

Active patient engagement can improve medication safety,^17^ but how these principles are experienced daily in home care and family caregiving remains unclear.^18^ While municipal home care provides most support, family caregivers often bear substantial, stressful responsibilities for medication management, which can require additional support.^16^,^19^ A meta synthesis of qualitative research^20^ suggests that medication management at home may become particularly challenging for older persons and their caregivers during transitions between hospital and home, due to fragmented information, communication barriers, disrupted continuity, and limited involvement in decision-making. Previous research has particularly focused on communication-related aspects, while broader medication experiences in everyday life and home settings appear less explored.^20^

As the older adult population grows, an increasing number will require support with medication management via home care services. By exploring the experiences of older persons and family members regarding medication management in home care, this study contributes knowledge that may support the development of safer and more person-centered home care services. This study aimed to explore the experiences and preferences of older adults living at home and their family caregivers regarding medication use and medication management support through home care. Specifically, it sought to address the following research question: What challenges, experiences, and preferences do older adults living at home and their family caregivers describe in relation to medication use and medication management support through home care?

## Materials and Methods

### Study Design

The study used a participatory design in which older adults served as co-researchers. These co-researchers were not home care service users themselves but were recruited based on age and relevant competencies (see Co-researcher engagement). The design of this qualitative study was grounded in co-research, which entails collaborating with participants in key aspects of the research process, including developing research questions and collecting and analyzing data.^21^,^22^ According to Littlechild et al,^22^ involving older co-researchers can improve data quality by enabling better communication, drawing on shared experiences, and creating a more comfortable interview atmosphere for participants. In line with these insights and inspired by Buffel’s work on engaging older adults as co-researchers,^23^,^24^ our study adopted a participatory design that recognizes older adults’ rights to be involved in generating knowledge that concerns their own lives and strengthens the relevance of the research by incorporating their perspectives on aging and community life.

The Consolidated Criteria for Reporting Qualitative Research guidelines^25^ were followed throughout the study design and reporting (Appendix 1) to ensure comprehensive and transparent reporting.

### Co-Researcher Engagement

The co-researchers were recruited via purposive and snowball sampling^26^ from a rural municipality in central Norway. Purposive sampling was conducted through managers in municipal home care services. These managers recommended suitable individuals aged over 65 years who were involved in promoting the well-being of older residents and had experience handling confidential information. The first author contacted 11 men and seven women by text message and phone. Three men agreed to participate as co-researchers. The fourth co-researcher was identified through snowball sampling, with one of the three initial co-researchers recommending him for the role. These four co-researchers, aged 70–75 years, were retired and had diverse working careers, including education, municipal administration, and the private sector. The co-researchers attended seven seminars led by the research team over 10 months. The seminars covered research ethics, qualitative interviewing, and data analysis. The co-researchers contributed to refining interview guides for older adults and family caregivers and conducted interviews with them. Before data collection, the co-researchers practiced interviewing each other using questions from the interview guide, and they reflected on how to foster communication, establish trust, and maintain an appropriate balance between closeness and distance in the interview setting. They placed particular emphasis on creating a safe, comfortable social atmosphere to ensure participants experienced the conversation as both natural and meaningful. The co-researchers actively participated in the data analysis (as detailed in the Data Collection and Analysis sections) and in the dissemination of findings, including contributing as co-authors in this article. Further details about their training and contributions will be provided in a separate methodological article. The section “Strengths and limitations” discusses how their involvement may have influenced the findings and methodological rigor.

### Research Context and Participants

In Norway, health policy prioritizes helping older adults to remain living at home for as long as possible.^27^ Home care services are publicly funded and free of charge. A common service in home care is medication management support.^28^ Medication management in Norway is defined as any medication-related task performed from the moment it is prescribed or ordered until it is given or, if relevant, discarded.^29^ Support levels vary from bi-weekly multidose drug dispensing (MDD), or pill organizer usage, to daily medication administration. Recipients may reside in private homes, municipally adapted housing, or residential complexes with 24-hour healthcare staff. Both health status and medication management may require support. Typically, municipal home care services provide this support. Some also have family caregivers who bear significant responsibility, which can be demanding and stressful and can necessitate additional help.^16^,^19^

While about 87% of older adults in Norway live in medium or large municipalities, nearly half of all municipalities are small, with fewer than 5,000 residents. These small municipalities have the largest proportion of residents aged ≥65 years and the greatest share of care service users in this age group, posing significant challenges for resource allocation and service delivery.^30^ The participating municipality in this project has fewer than 5,000 inhabitants and is situated in a large, geographically extensive area with a dispersed settlement pattern typical of Norway. The municipality features a municipal center offering various public services, and home care services are organized into two zones.

Older adults were purposively sampled—selected for their ability to provide in-depth insights into the phenomenon under study^26^ Eligible participants were identified based on predefined inclusion criteria and were informed by municipal staff, who asked whether they consented to being contacted by the researchers. The inclusion criteria were age of ≥65 years, at least one individual decision on medication management support, understanding of the study’s nature and implications, and provision of informed consent. Older adults with cognitive impairments were excluded. The first author received contact information for individuals who had expressed interest in participating. The co-researchers contacted potential participants by telephone. The co-researchers provided both written and verbal information before obtaining consent. A total of 12 older adults were approached, with 11 agreeing to participate in interviews. The older adults who consented were also asked whether they had a family caregiver who could be contacted. Six older adults permitted the co-researchers to invite their caregivers to participate. A family caregiver was defined as a relative providing support, such as a spouse, son, or daughter. Six family caregivers were approached by phone and consented to participation.

### Data Collection

Semi-structured interviews were conducted in 2024 and audio-recorded by the co-researchers, who interviewed two to eight participants each, including both older adults and family caregivers.

The interviews followed semi-structured interview guides.^31^ The interview questions were informed by previous research and aligned with the study’s aim. The interview guides (Appendix 2) were initially developed by the research team and subsequently collaboratively revised together with the co-researchers. Separate guides were developed for older adults and family caregivers, respectively, to capture their distinct experiences and perspectives while facilitating dialogue about medication use and management in everyday life, knowledge of personal medication routines, experiences with home care services, and challenges and preferences related to safe, person-centered medication management.

Each interview began with a brief introduction to the study, including information about audio recording, de-identification, informed consent, and participants’ right to withdraw at any time without consequences. The interviews were conducted in a conversational style.

Before data collection, the co-researchers tested and refined the interview guide by interviewing one another. After completing their first interview, the co-researchers were invited to a debriefing session and a group interview facilitated by the research team. This provided an opportunity to share both challenges and aspects they felt had gone well. Before debriefing, the first author listened to the interview recordings and guided on issues such as leading questions, interviewer impatience, and unintentional attempts to take control of the conversation, as well as tips to increase interview length. The interview guide was revised after the debriefing session based on feedback from the co-researchers. Changes included reordering questions, adjusting some language, and adding a few sub-questions.

All interviews were conducted in person within participants’ homes, per their preference. The co-researchers spent up to 3 hours in participants’ homes. They were served coffee and engaged in long, unrecorded conversations with participants. The audio recordings ranged from 15 to 40 minutes and were stored in a secure data storage system with access restricted to the first and last authors. The transcripts were automatically generated by the storage system and subsequently reviewed, de-identified, and quality-assured by the first author.

### Data Analysis

**Figure 1:**
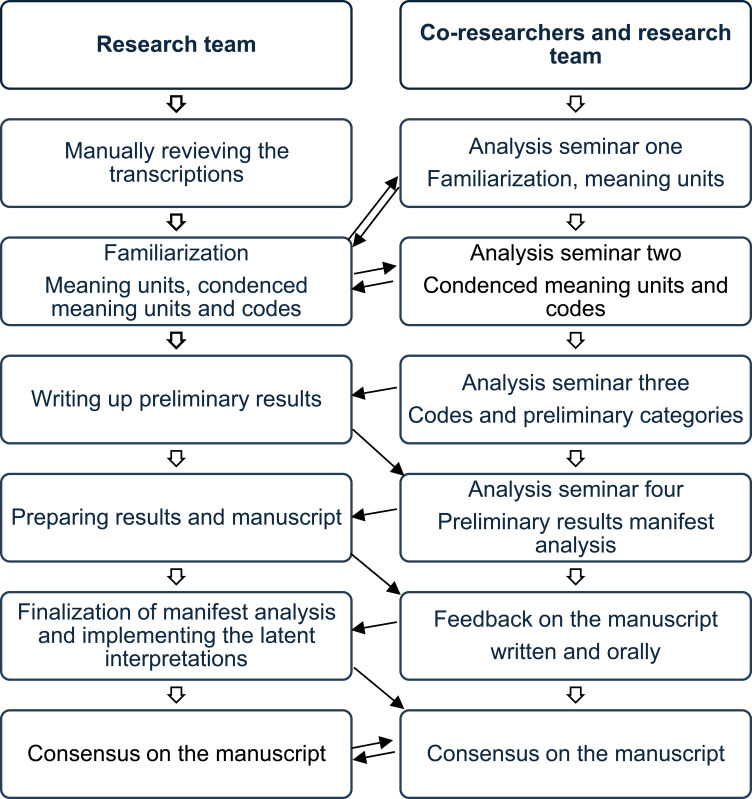
Schematic representation of the research team’s analysis and manuscript development process, including their interactions with co-researchers during data analysis.

Data were analyzed via inductive qualitative content analysis as described by Graneheim and Lundman^32^ and Graneheim et al^33^ Initially, the analysis focused on the text’s manifest content. This approach enabled close engagement with the data and facilitated the identification of concrete, readily recognizable content. The content analysis also allowed interpretation at a more abstract level to capture an overarching theme.^33^ During analysis, we continuously assessed the richness, variation, and conceptual depth of the material. Rather than aiming for saturation in the sense of “no new information,” we drew on a meaning-oriented understanding of sufficiency, considering whether the interviews provided sufficiently nuanced and diverse accounts to support in-depth interpretations of participants’ experiences in relation to the study aim.^34^ Co-researchers also contributed to assessing the richness and relevance of the material, drawing on experiential knowledge when discussing whether important dimensions of participants’ experiences had been sufficiently explored. The process of analysis and interaction between the co-researchers and research team is schematically outlined in Figure 1.

The analysis process followed an iterative and collaborative approach involving both the research team and the co-researchers (Figure 1). The process began with the first author manually reviewing the audio-taped interviews and automated transcriptions. The research team then individually familiarized themselves with the entire dataset, identifying meaning units, condensed meaning units, and preliminary codes. These steps, as illustrated in the upper part of Figure 1, were conducted manually and in the qualitative analysis software NVivo 15.3.2.

After this initial familiarization, the research team and co-researchers collaborated in four structured analysis seminars. In seminar one, co-researchers read the transcripts and identified meaning units in groups consisting of two co-researchers and one to two researchers. In seminars two and three, the co-researchers used Post-it notes to sort condensed meaning units and codes into preliminary categories, thereby actively contributing to the analysis. Before seminar four, the research team refined the categories and prepared preliminary results. During seminar four, co-researchers provided further input on the developing manifest analysis and offered feedback on the written summaries.

Throughout the process, the analysis was iterative rather than linear, involving continuous movement between the whole and parts of the data. The research team repeatedly returned to the transcripts, allowing them to confirm and enrich interpretations with contextual insights from the interview process. Following joint discussions and an overall interpretation of the latent meaning within the categories, a recurring latent pattern^33^ and overarching theme was identified: between feeling safe and being safe: balancing autonomy and support in medication management at home. This theme captured a dual dimension of person-centered, safe medication management practices among older adults and family caregivers.

### Ethical Considerations

This study was conducted in accordance with the ethical Principles of the Declaration of Helsinki.^35^ All participants received both verbal and written information about the study’s purpose and the co-researcher’s role and were allowed to ask questions before providing informed consent. Explicit consent was obtained for participation in the study and audio recording of the interviews. Participants consented to the collected data being de-identified and used for research dissemination, including publications and conference presentations. All reported excerpts have been fully de-identified to ensure that no participants can be identified.

All audio recordings and related data were securely stored and scheduled for deletion within a specified timeframe. To ensure confidentiality, each participant was assigned a unique identification number, and all data were de-identified accordingly. The study was assessed by the Norwegian Agency for Shared Services in Education and Research for compliance with data protection regulations (ID: 759436). Furthermore, the Regional Committee for Medical and Health Research Ethics North evaluated the study protocol and determined that formal ethical approval was not required (ID: 629685).

Co-researcher involvement raised several ethical challenges. A key consideration was that older adults and their relatives had been informed that co-researchers would collect their data. Moreover, the co-researchers were members of the same local community as participants, which meant that interviewers and interviewees might be prior acquaintances. This could have influenced the interview situation both positively and negatively. Initially, the researchers assigned participants to the co-researchers to establish contact and conduct interviews. Simultaneously, the co-researchers were consulted during allocation to identify potential conflicts of interest or prior relationships that might pose challenges. In one case, the original allocation was adjusted because an existing acquaintance was considered to be better for the person being interviewed.

Research ethics and secure handling of personal data were emphasized during the co-researchers’ training. A formal contract was established between the co-researchers, as employees, and Nord University, as the employer, to underscore their role as researchers. The co-researcher role was thus formalized through a written agreement. The co-researchers signed a confidentiality agreement, received a salary for their contributions, and had their travel expenses covered by the project.

Ethical challenges were also discussed regularly with the co-researchers in the seminars, both during preparation and after the interviews. The co-researchers were highly attentive to these issues and raised numerous potential dilemmas, which were collectively explored and reflected upon.

### The Use of Artificial Intelligence

The interviews were initially transcribed by AI (OpenAI Whisper V3) using “Nettskjema,” A service provided by the University of Oslo. The transcripts were then manually reviewed and corrected for errors by the first author. Microsoft Co-Pilot, an AI recommended by Nord University, was used as a discussion partner and for proofreading. Grammarly Pro was also employed to improve language accuracy.

## Results

### Participants

A total of 17 participants were included in the study. Table 1 shows their background information.

**Table 1:** Overview of the Participants

	Older Adults	Family Caregivers
n (%)	11 (65)	6 (35)
Median age (min–max), years	90 (71–94)	60 (55–78)
Men, n (%)	4 (36)	2 (33)
Residents		
Private homes or apartments, n (%)	5 (45)	
Residential care, n (%)	6 (55)	

Among the six family caregivers, there were one spouse, three daughters, and two sons. Two caregivers were healthcare professionals. All but one family caregiver lived in the same municipality as the older adult they cared for. One caregiver declined to be audio-recorded; in this case, the co-researcher produced a written summary of the interview. The spouses of two older adults were present during the interviews; these spouses were not interviewed individually. They received the offer but wanted to be present for their spouse’s interview.

**Figure 2:**
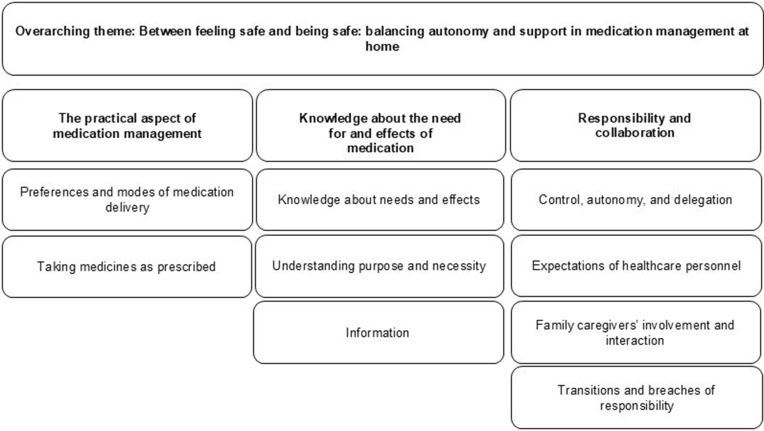
Overview of the findings: The overarching theme, categories, and subcategories.

The analysis yielded three main categories and nine subcategories (Figure 2).

### Transparency

For transparency, Table 2 provides examples of how meaning units were mapped to codes, subcategories, and categories, illustrating the logic of the analytical process. The findings reflect the experiences of both older adults and family caregivers.

**Table 2:** Examples from the Analysis

**Category: Practical Aspects of Medication Management**		
Subcategory	Code	Meaning unit
Preferences and modes of medication delivery	MDD vs. pill organizer	“My mom’s system with the multidose is actually quite reassuring—it’s really helpful because it reduces the chance of her taking too much at once, which can sometimes happen with those traditional pill organizers or boxes.” (Family caregiver)
Taking medicines as prescribed	Errors and irregularities	“We observed that she was using prednisolone in a way that seemed more like a substitute for paracetamol, and it was clear that it was not good for her. That’s when our concern deepened, and we thought”, ‘This can’t go on.» (Family caregiver)
**Category: Knowledge about the need for and effects of medications**		
*Subcategory*	*Code*	*Meaning unit*
Knowledge about effects and side effects	Effects and side effects	“Some medicines make me feel very tired in the morning because I take them in the morning. However, I might be partly to blame, as I’ve switched between taking them in the evening and the morning.” (Older adult)
Understanding purpose and necessity	Concerns or confusions	“I’m not entirely sure if I need all the medicines I’m taking. I have several different ones, and while I understand why I take some of them, I’m a bit unclear about the reasons for others.”(Older adult)
Information	Information gaps and contradictory information	“I often wonder if I really need the medication I am taking. It’s something I think about quite a bit. You see, I visit my GP [general practitioner], and he prescribes medication, but then I go to the hospital, and they tell me, you do not need that one anymore”.» (Older adult)
**Category: Responsibility and collaboration in medication management**		
*Subcategory*	*Code*	*Meaning unit*
Control, autonomy, and delegation	Autonomy and independence	“I prefer not to reveal that I take [pain] medications. I don’t want others to know what I am using. I would have liked to keep the painkillers for myself, as I did before. That would have made me feel safer.” (Older adult)
Expectations of healthcare personnel	Follow-up and system reliability	“I wish there was more follow-up regarding my medications—and not just receiving the entire pill roll at once, at least once in a while.” (Older adult)
Family caregivers’ involvement and interaction	Family caregivers’ role in medication management	“Whenever I visit, which happens fairly often, I make an effort to stay attentive and observe carefully.” (Family caregiver)
Transitions and breaches of responsibility	Being rushed to/from the hospital	Things then happened very quickly, I will tell you [about the discharge from the hospital]. I was practically rushed out, almost pulled away… I kindly asked if I could stay until the next day, but that was not really possible. There were so many other patients there. When I got home that evening, I realized the electronic medical records system was not working yet, so they did not know which medications I was supposed to receive. I almost started to panic at that moment. (Older adult)

### Between Feeling Safe and Being Safe: Balancing Autonomy and Support in Medication Management at Home

The overarching theme reflects a tension in the findings between feeling safe as an inner sense of being cared for and being safe as external protection from harm. While routines and procedures within home care services are designed to promote patient safety, they must also accommodate the needs expressed by older adults and their family caregivers, who emphasized that knowledge, involvement, and a sense of control are essential to their experience of feeling safe.Addresing both feeling safe and being safe therefore require an approach that is sensitive to the individual preferences, capacities, and circumstances of each older adult.

### Practical Aspects of Medication Delivery

#### Preferences and Modes of Medication Delivery

This subcategory covers the participants’ descriptions of practical elements such as preferred delivery methods, differences between MDD and pill organizers, and resistance to generic substitution.

Most older adults received their pre-packaged medications from home care services, either in a weekly pill organizer or through bi-weekly MDD. Some also received single doses directly from home care staff, while others managed their own as-needed medications. The participants held differing views on which mode of medication delivery was best. Some participants considered MDD systems to be the simplest and safest solution, and others preferred a weekly pill organizer because it was easier to manage. Some reported difficulty opening MDD bags, and the family caregivers mentioned that tablets were occasionally dropped on the floor. Additionally, some medications were described as so small that they were easily lost when opening bags, with the caregivers occasionally finding tablets under the sofa. However, the municipality’s practice appeared to favor MDD, and some older adults reported feeling limited freedom of choice.

One older adult who received weekly delivery of a pill organizer explained that she would likely switch to MDD:

Currently, I use a weekly pill organizer. On Wednesday morning, I take the last dose, and the home care nurses deliver a new one at 1 p.m. They mentioned a different system to replace the organizer, but I don’t know whether that’s a positive development. I have no idea. They’ve been adjusting medication dosage—more of some, less of others…—so it is not that simple to switch to something new. The pharmacy then has to set it up and organize it. But you know, I’m not sure I’m going to keep this new routine, so maybe it’s better if the nurses handle it as they do now.

Regardless of the administration method, most participants expressed trust in the system, but several older adults wanted to know they had the correct supply of medications and a fixed storage place. They stated that having a clear overview and control of their medication was essential for feeling safe and that they would have been “completely lost” in a certain challenging situation without that control.

Among the participants, generic substitution was a recurring concern that led them to feel uncertain or skeptical when given medications they did not recognize. One older adult explained, “It makes me somewhat skeptical because there could be side effects. I know that many people prefer the medication prescribed by their doctor.”

Receiving a generic substitution was especially concerning if they had not been informed about the change, with one participant saying, “And then I had to ask, ‘I’m not taking this. What is it? I don’t have a pill like that.’ They explained it to me afterward, but they could have done that earlier”.

#### Taking Medications as Prescribed

This subcategory offers an overview of medication-taking routines, adherence, nonadherence, errors, and irregularities.

The family caregivers emphasized the importance of administering medications at the correct times and doses. Some caregivers were unsure whether medications were taken as prescribed, while others had observed missed doses or incorrect use. Conversely, medication use appeared to be a habitual, automatic routine for the older adults. For instance, one older adult took sleeping medication just in case when waking at night, even if they had already fallen asleep without it.

The older adults generally expressed an almost automatic trust that their medication regimen was appropriate, but some also expressed uncertainty and doubt. One family caregiver said:

I try to keep an eye out, especially when it comes to pain, because my mother is reluctant to take pain medication. She uses paracetamol, but she is very cautious of taking too much, so she splits it in half. So, she is not taking any chances.

Some older adults reported making independent choices about certain medications. One participant who had antibiotics to use as needed emphasized the importance of independently managing antibiotics when experiencing a fever and other signs of infection to avoid worsening illness, as previous treatment delays resulted in hospitalization and severe infections.

One family caregiver shared that their siblings believed their mother was not adhering to the prescribed antidepressant regimen:

She doesn’t always want to take the medications. She kind of waits to take them, and then she figures—maybe I don’t need them today… She says they make her feel “foggy” or that she doesn’t need them. We’ve tried to persuade her, as have the healthcare professionals, because we see that she becomes more cheerful when taking them.

### Knowledge About the Need for and Effects of Medications

#### Knowledge About the Effects and Side Effects of Medications

This subcategory covers the effects and side effects reported by the older adults or observed by the family caregivers. One older adult said, “The effect…—I don’t know if the medicine works at all.” Another stated, “I don’t really notice any difference if I take the medication or not.”

Some older adults reported side effects, such as tiredness or sleepiness. Some side effects led them to stop taking the prescribed medicine or refuse it. An older adult shared,

They changed my medication at the hospital, but had to switch back because my blood pressure dropped too low. I kept fainting, and I don’t want a life like that.

There were also examples of older adults not reporting symptoms that were later identified as side effects following medication changes. The family caregivers who recounted these episodes emphasized the importance of both the older person and their family members having information about what to react to and when.

#### Understanding Purpose and Necessity

This subcategory describes trust in prescriptions and reveals the participants’ understanding of prescribed medicines. It also covers the participants’ perspectives on why their medication was necessary and addresses their concerns. One older adult said, “I am not sure whether I need the medicines I am taking. I assume I’ve been given the medication to help me function…”

Some older adults also wondered whether the medicines they took “affected each other.” When asked whether they felt safe that the medicine they received was appropriate for them, one older adult said, “I don’t feel completely safe, but… that’s how it is.” Another older adult uttered, “I trust that those who give me my medicine provide me with the medicine I need.” One of the family caregivers said, “This generation still tends to agree with anything the doctor suggests because they have great respect for the doctor.”

#### Information

This subcategory covers discussions with the doctor about medications, including changes, information gaps, contradictory information, a lack of medication dialogue, and medication review.

Few older adults discussed medications with their doctor. The impression was that they visited their general practitioner (GP), were prescribed medication, and took it as directed, with minimal discussion about why they needed the medicine or what side effects to be aware of. One older adult mentioned, “It’s like I’ve been prescribed medication, but I haven’t actually discussed it with my doctor.” Another said,

No, we haven’t really discussed medicines, except when I was there and received the prescription. That’s why I don’t understand why I’m taking all the medicines… or I do understand it for some of them…

When asked how they learned about their medicines, several participants mentioned obtaining information from prescription labels and package leaflets. However, they found navigating the leaflets challenging partly because the text appeared small and nearly unreadable. Only a few reported actually reading them.

Both older adults and family caregivers reported receiving minimal unsolicited information about medications, and several expressed a desire for more information and guidance. The family caregivers shared these concerns and called for a more thorough review and evaluation of medication regimens for older adults living at home, especially when their medications changed. Some family caregivers noted that prescriptions remained unchanged over time without regular reassessment of their necessity. Both older adults and family caregivers expressed that explanations and more information would make a difference. They emphasized that information should be repeated, as older adults might have hearing difficulties or forget what was said during an appointment with the GP. A desire to receive written information in addition to verbal explanations, such as an overview from the GP or the home care service, was also mentioned.

The older adults acknowledged receiving answers to their specific questions, either from home care staff or from their GP. Some admitted they should have been more active in seeking information and said they might have asked for it. Others described being highly proactive:

I had to ask. I sat in the hallway in the assisted living building, waiting for them, the staff. And then I said, ‘You have to come with me and see if I’m doing this correctly.

### Responsibility and Collaboration in Medication Management

#### Control, Autonomy, and Delegation

This subcategory covers the older adults’ and caregivers’ views on control, autonomy, independence, delegation, questioning, privacy, dignity, future needs, and oversight maintenance.

While some older adults wanted to keep control, others considered it acceptable for the home care service to take over managing their medicines. One older adult who was severely ill for a period said, “And after that, home care has taken over medication management because I didn’t have the strength to manage prescriptions and such.” Other older adults indicated they had not fully explored the medicines they were taking and why because home care services handled them.

The family caregivers also emphasized that they wanted arrangements that could support the older adults’ independence and coping. However, they also mentioned attempting to monitor for any changes that might indicate deviations from the correct medication, dose, or timing. The family caregivers noted that vulnerability to changes in routines, staffing, or the older adults’ health status was a significant source of uncertainty, reducing the likelihood of maintaining the older adults’ independence. Additionally, they said that “this work for now” but acknowledged that there might come a time when control would need to be handed over to others.

One family caregiver said:

I try not to interfere too much; my philosophy is that mum should take care of what she can do herself and have the chance to fix things on her own. It appears she feels she is aging, needing more support, especially with medications, which she desires the home care service to manage. However, she also wants control over her medicines and to decide when to take them… I believe that home care services will manage the medicines eventually, but it’s ultimately my mom who figures it out.

When asked about whether they felt that they had any influence over their medication, one older adult said, “No, I just have to accept it because… I rarely used medicine before. But when you first encounter that situation, you just have to… take it.”

Among the older adults who more clearly asserted independence, prior negative experiences or adverse events appeared to play a decisive role. They had learned the importance of standing up for themselves. One older adult described an error in medication management after a hospital stay, in which the home care nurses had not received the correct information:

When they [home care nurses] came, I had already sorted it out and taken the correct morning dose because I just had to look into it and manage it myself. You must be capable yourself. That was necessary… That was an ugly misunderstanding, but it didn’t affect me much because I knew what I was supposed to take. However, if it had happened to someone who wasn’t in control, it would have been a serious problem.

The family caregivers reported that the older adults were accustomed to and felt safe with their existing routines and that changes in general were increasingly challenging as they aged. One of them said, “They [doctors] increased the dose on one medicine, and that made her worse, but she kept it to herself.”

#### Expectations of Healthcare Personnel

This subcategory contains information about the help the participants received, as well as their relationships with healthcare personnel, expectations, and experiences regarding follow-up and responsibility.

Several participants mentioned MDD during the interviews, and some also requested more follow-up from the home care nurses “than just receiving the roll.” One participant was markedly satisfied with having received a medication review during a hospital stay. No participant experienced this in the municipality.

The older adults expressed great trust in the home care personnel and were grateful for the support they received with their medications. The participants expected the home care staff to have a good overview of medications and to maintain communication with the GP. Several said they assumed that necessary information was exchanged between healthcare professionals and that they received answers when they asked questions. The home care nurses were specifically mentioned as important points of contact. The GP was more difficult to contact.

Experiencing that adjustments were made or that wishes to self-administer sleeping pills or pain medication were accommodated was mentioned as important. It was also important that the older adults felt they were met by caregivers who saw and respected them and took the time to provide personal care. One older adult recounted an episode of acute illness:

And she sat there for nearly 1 hour. She talked with me and stroked my hand. That little hour she sat there, talking to me and stroking my hand, was much better than 30 pills, honestly.

However, experiences with medication errors, failures in information transfer, or generic substitutions could influence future trust. Those who had such experiences were more vigilant and reported that it was essential for them to monitor their own medications.

The family caregivers also expressed general trust in the service but noted they were uncertain about what the home care nurses actually did or monitored during visits to the older adults.

#### Family Caregivers` Involvement and Interaction

All family caregivers lived close to the older adults, and one was a spouse. Although not all family caregivers provided concrete, practical support with daily medication management, they still appeared to serve as a safety net. The older adults often said they wanted and found it reassuring to have family caregivers accompany them to GP appointments and handle things if anything about their medications was unclear. One of them said, “If I don’t catch it all, my son will tell me on the ride home.”

Several examples were given of family caregivers accompanying the older adults to medical appointments, helping them ask questions and remember the information, thereby fostering a sense of feeling safe. Several family caregivers mentioned that they attempted to monitor for any changes that might indicate deviations from the correct medication, dose, or timing.

No family caregivers reported having any agreed-upon responsibility to assist the older adults with medication management. Additionally, they stayed attentive without seeming intrusive. One family caregiver mentioned that because she worked in healthcare, she had extensive insights into both pharmacology and how healthcare professionals work. It struck her during the interview that other family caregivers did not have this advantage. She said:

If I check the multidose pack and see that something is wrong, I contact the home care service. I try to stay a bit on the sidelines… because my philosophy is that my mom should handle what she can herself… If there are changes in medication, the patient is always informed, but the relatives are not. There is definitely room for improvement in the collaboration between the home care service and family caregivers.

This was confirmed by several other family caregivers, who felt that both family caregivers and healthcare personnel needed to improve their communication and coordination with one another.

The family caregivers reported that it would have been easier for them to contribute with observations or support if they had sufficient information about medication use. Those with a healthcare background considered this a major advantage, as they had both general knowledge about medications and a clearer understanding of the scope and limitations of home care services.

#### Transitions and Breaches of Responsibility

Several participants provided examples of situations in which different types of transitions were the source of errors and uncertainty. Transitions between hospital and home were highlighted as critical due to the risk of important information being lost. Other organizational transitions, such as holiday periods with increased use of temporary staff, raised similar concerns; the participants worried about whether correct medication information would be passed on.

One older adult discharged from the hospital before she felt ready experienced that the information about her medicines was not transmitted to home care services. The lack of information and uncertainty surrounding her medicines became an additional burden at a time when she was already seriously ill and vulnerable. She explained, “It affected me a lot because I could not handle any more stress after an incident like that, so I felt helpless at the time.”

The participants also pointed to transitions between different administration systems, such as pill dispensers and MDD. One family caregiver said, “It creates uneasiness, uncertainty, and they [older adults] become scared.”

## Discussion

This study explored the experiences and preferences of older adults living at home and their family caregivers relative to medication use and medication management support through home care. The analysis revealed three main categories—practical aspects of medication management, knowledge about the need for and effects of medications, and responsibility and collaboration—all of which illuminated how the participants navigated medication use in everyday life. Across these categories, an overarching theme, between feeling safe and being safe: balancing autonomy and support in medication management at home, captured the tension between safety as externally defined protection from harm and safety as an internal sense of being cared for. This distinction between safety as defined by the healthcare system and safety as experienced by older adults and family caregivers is central to interpreting the findings. Importantly, this tension resonates with core assumptions in person-centered practice, where safety is not limited to technical correctness but is understood as emerging through relationships, involvement, and shared understanding between professionals, older adults, and family caregivers.^9^

While home care routines and procedures are designed to ensure safe medication practices, both older adults and family caregivers described playing a more passive role than they preferred, often accepting what was offered while withholding questions or preferences for more individualized support. These findings indicate that achieving both feeling safe and being safe requires person‑centered approaches that actively foster involvement, knowledge, and sense of control in home medication management. Within the framework of McCormack and McCance,^9^ this suggests that person-centered processes such as working with patients’ beliefs and values, shared decision making, and genuine engagement were only partly realized in everyday medication practices.

Most current safety measures in medication management are grounded in a system-oriented understanding of patient safety, emphasizing standardization, risk reduction, and error prevention.^36^ The participants acknowledged the value of these measures but also described their sense of safety as depending on more than correct procedures. They mentioned feeling safe relative to whether they experienced attentiveness, recognition, and responsiveness from healthcare personnel. This reflects a distinction between safety as “being safe,” understood through compliance with standardized procedures, and safety as “feeling safe,” which depends on relational and contextual factors. Such distinctions have also been discussed in patient safety literature contrasting system-focused safety approaches with perspectives that emphasize patients’ lived experiences, relational aspects of care, and adaptive practices in everyday healthcare delivery.^37^ Prior research similarly shows that person-centered care enhances both perceived safety and well-being by emphasizing relational aspects of care rather than solely technical correctness.^9^,^13^

The tension between “being safe” (procedural correctness) and “feeling safe” (relational reassurance and autonomy) was depicted in all interviews. The participants who felt informed, involved, and in control described a stronger sense of safety even when practical challenges existed. Conversely, those who felt excluded from decisions or poorly informed expressed uncertainty and mistrust even when procedures were followed.

Limited understanding of medication purpose, expected effects, and reasons for changes contributed to uncertainty among many participants. Several reported rarely discussing medications with their GP, and generic substitutions frequently created confusion. The lack of explanation diminished trust and could lead to passive acceptance rather than active participation. Therefore, knowledge gaps reduced the ability to evaluate one’s own situation and undermined the sense of control—a crucial component of perceived safety. This supports previous findings indicating that accessible, tailored medication information strengthens both empowerment and adherence.^13^,^38^ For the family caregivers, insufficient information often meant they felt compelled to monitor the older adults’ medications more closely, blurring role boundaries and creating tension between trust and vigilance, consistent with previous reports.^39^

Shared decision-making is central to person-centered medication practices.^40^ However, many older adults in this study reported having few opportunities to ask questions, express concerns, or participate in decision-making (eg, regarding administration methods). Power differentials in patient–provider relationships were evident; several participants assumed a subordinate role. These findings suggest that limited involvement may reflect structural and relational conditions rather than individual preferences alone, highlighting the importance of proactively inviting participation rather than interpreting silence as satisfaction.^9^,^41^

Individual preferences also varied: Not all participants wanted extensive involvement, which aligns with earlier work.^40^ Therefore, person-centered care requires flexibility, recognizing both those who desire active involvement and those who prefer to delegate.^9^

Nonetheless, even among the participants who preferred a passive role, a lack of involvement influenced feelings of safety. Being included did not necessarily mean making all decisions, but rather having the opportunity to understand, ask questions, and feel respected.

Standardization—such as the widespread use of MDD—was intended to enhance safety. However, it sometimes conflicted with the older adults’ desire for control. For some, switching between medication delivery systems (pill organizers vs. MDD) disrupted routines that worked well for them. Others perceived standardized routines as inflexible and insufficiently tailored.

The distribution of roles among the home nursing services, older adults, and family caregivers was unclear. From a system level perspective, such ambiguity reflects gaps in the care environment, where unclear responsibilities and fragmented communication can undermine both involvement and safety, despite professional intentions to provide high quality care.^9^,^42^ This may have led to uncertainty and reduced involvement among the participants. They were uncertain about what was performed and who was responsible for medication management. Both older adults and family caregivers trusted that healthcare personnel had an overview of the medications and would alert the GP if anything was amiss. This sometimes led to hesitation to intervene when errors were suspected because they felt healthcare personnel had the same information as they did. This lack of clarity challenged decision-making and may have increased the vulnerability to mistakes.

A reduction in perceived control often meant a reduction in perceived safety. The participants who retained some degree of involvement, even within standardized frameworks (eg, being able to open the dispenser themselves or understanding the system), expressed greater confidence.

Transitions between care levels were considered moments of heightened vulnerability. The participants who had experienced discrepancies or errors during transitions became more vigilant and felt a need to double-check medications. Both older adults and family caregivers were often uncertain about who was responsible for what—home care, the GP, or the patient themselves. This lack of clarity challenged their sense of control and could undermine trust in the system.

The findings echo earlier research showing that fragmented communication and unclear responsibilities negatively impact care continuity and patients’ sense of safety.^14^,^42^

Collectively, the findings point to several practical implications for improving home medication management for older adults. Accessible, tailored information about medications—including purpose, expected effects, side effects, and reasons for changes—should be consistently provided, particularly during transitions such as hospital discharge or treatment adjustments. Regular follow-up and structured medication reviews are essential to address evolving needs and ensure safe use. Older adults and family caregivers should be given opportunities to influence how support is organized, including choices related to routines and administration, while clear roles, responsibilities, and communication pathways across providers can enhance predictability and coordination. Supporting autonomy requires recognizing diverse preferences for involvement and enabling control where desired. Finally, safe medication support benefits from the involvement of personnel who are familiar with patients’ history, preferences, and everyday context. At a system level, improvements in person-centered medication management require not only informational interventions but also organizational conditions that support continuity, dialogue, and professional discretion, thereby enabling person-centered processes to unfold in practice.^9^,^43^

The findings also indicate the need for more collaborative approaches to service development. Several challenges identified—limited involvement, unclear responsibilities, and tensions between standardization and individual preferences—are not easily addressed through traditional top‑down service improvements. Co‑creative and participatory design approaches, in which older adults, family caregivers, and home care personnel collaboratively identify challenges and develop solutions, may offer a more effective way to align system‑level practices with users’ everyday experiences. Such approaches have been shown to strengthen communication, ownership, and relevance of care practices, particularly in complex, multi‑actor systems such as home care.^44^,^45^

### Strengths and Limitations

This study was conducted in a small municipality with a dispersed settlement pattern, which is common in Norway. Although the findings reflect the experiences of a relatively small number of older adults and family caregivers, they are consistent with the aim of qualitative research, which is to provide rich, detailed insights rather than generalized conclusions. Transparency in the description of the study context, sampling, interview procedures, and participant characteristics, together with a careful presentation of illustrative quotations that allow readers to follow the logic, coherence, and credibility of the analysis, supports the transferability of the findings.

A qualitative descriptive design with a participatory approach was deliberately chosen to explore the participants’ experiences and strengthen older adults’ involvement. The co‑researchers played an important role by refining the interview guide and adding perspectives that broadened the research team’s viewpoint. Their involvement likely influenced both data collection and interpretation. Through their experiential knowledge, they may have facilitated trust and recognition during the interviews, encouraging participants to elaborate on experiences that might otherwise have remained implicit. At the same time, shared experiences and perspectives may also have influenced what was explored, emphasized, or taken for granted during interviews and analysis. The co-researchers’ diverse backgrounds enriched the discussions, while the broader research team contributed complementary expertise in home care practice, participatory design, social education, audiology, and leadership, supporting a reflective and multifaceted analytic process.

Municipal staff assisted with screening and recruitment, and we requested a broad representation of residents receiving medication support. The participants had to be 65 years of age or older, understand the study, and provide informed consent. Consequently, older adults with cognitive impairments were excluded. This exclusion represents a limitation, as cognitive challenges are common among individuals receiving medication support and may influence needs related to communication, safety, and daily medication management. Individual semi‑structured interviews were chosen to allow open discussion of personal and sometimes sensitive topics related to medicines and health; focus groups were considered less suitable for this purpose.

Another limitation is that the co‑researchers were young older adults and were not required to be home care recipients themselves. Including co‑researchers with direct experience of receiving medication support might have enabled even deeper engagement with the findings. However, several had experience as family caregivers, which added relevant insights. Additionally, variations in interviewer experience—four individuals conducting two to eight interviews each—may have influenced data consistency and depth. Therefore, differences in interview length and style should be acknowledged.

## Conclusion

This study demonstrates that safe home medication management for older adults involves both procedural correctness (being safe) and a subjective sense of feeling safe, rooted in knowledge, involvement, and supportive interactions. Although home care services aim to enhance safety through standardized routines, many older adults and family caregivers experience limited opportunities to actively engage in decision-making, ask questions, or influence how support is provided. This often leads to a passive role, diminishing their sense of control and safety.

Hence, strengthening medication safety in home care requires a more person‑centered approach that recognizes individual preferences for autonomy, ensures accessible and tailored information, and clarifies responsibilities across care providers. Supporting meaningful involvement—whether through active participation or informed delegation—can enhance both “being safe” and “feeling safe.”

Future research and practice should explore how person‑centered medication practices can be integrated into existing home care structures through co‑creative and participatory design approaches. Involving older adults, family caregivers, and frontline healthcare personnel in direct dialogue about needs, challenges, and feasible solutions may enable the development of practices and systems that are clinically sound and experientially safe.

## Abbreviations

GP, general practitioner; MDD, multidose drug dispensing.
